# Statistical Regularities Attract Attention when Task-Relevant

**DOI:** 10.3389/fnhum.2016.00042

**Published:** 2016-02-09

**Authors:** Andrea Alamia, Alexandre Zénon

**Affiliations:** Institute of Neuroscience, Université catholique de LouvainBruxelles, Belgium

**Keywords:** visual attention, eye tracking, statistical learning, implicit learning, selective attention

## Abstract

Visual attention seems essential for learning the statistical regularities in our environment, a process known as statistical learning. However, how attention is allocated when exploring a novel visual scene whose statistical structure is unknown remains unclear. In order to address this question, we investigated visual attention allocation during a task in which we manipulated the conditional probability of occurrence of colored stimuli, unbeknown to the subjects. Participants were instructed to detect a target colored dot among two dots moving along separate circular paths. We evaluated implicit statistical learning, i.e., the effect of color predictability on reaction times (RTs), and recorded eye position concurrently. Attention allocation was indexed by comparing the Mahalanobis distance between the position, velocity and acceleration of the eyes and the two colored dots. We found that learning the conditional probabilities occurred very early during the course of the experiment as shown by the fact that, starting already from the first block, predictable stimuli were detected with shorter RT than unpredictable ones. In terms of attentional allocation, we found that the predictive stimulus attracted gaze only when it was informative about the occurrence of the target but not when it predicted the occurrence of a task-irrelevant stimulus. This suggests that attention allocation was influenced by regularities only when they were instrumental in performing the task. Moreover, we found that the attentional bias towards task-relevant predictive stimuli occurred at a very early stage of learning, concomitantly with the first effects of learning on RT. In conclusion, these results show that statistical regularities capture visual attention only after a few occurrences, provided these regularities are instrumental to perform the task.

## Introduction

One of the central functions of the human brain is the ability to predict the surrounding dynamics and to optimize interactions with the environment (Clark, 2013; Little and Sommer, 2013). Learning contingencies and regularities is a multi-faceted and elaborated mechanism that allows the brain to perform predictions and optimization (Dayan et al., 2000; Kruschke, 2003; O’Brien and Raymond, 2012). Attention is regarded as an important mechanism involved in reducing perceptual uncertainty but its role in learning remains controversial (Gottlieb, 2012). On the one hand, a model proposed by Pearce and Hall (1980) suggests that unpredictable and surprising cues capture attention more than predictable ones, supposedly because they provide new information about the environmental contingencies. This view is supported by experimental studies that have showed that attention gets preferentially allocated to conditioned stimuli with uncertain outcomes (Hogarth et al., 2008). On the other hand, an alternative model (Mackintosh, 1975), which has also received recent experimental support (Kruschke, 2001; Le Pelley et al., 2011), suggests the opposite view, arguing that predictability attracts attention and that this early attentional capture would be instrumental in learning. Lately, an hybrid model integrating the two theories has been proposed in order to conciliate these controversial experimental findings, postulating the co-existence of two distinct attentional systems, namely a controlled system processing the most unpredictable stimuli in order to learn the dynamics of the environment and an automatic one, exploiting information already acquired and focusing on the stimuli essential to perform the task at hand (Le Pelley, 2004; Pearce and Mackintosh, 2010). A similar reading of the role of attention in learning has been provided by Dayan et al. (2000), who considered the Pearce–Hall model (Pearce and Hall, 1980) as appropriate to explore and learn regularities in a new environment whereas the Mackintosh model (Mackintosh, 1975) would drive behavior during the routine execution of a task.

It is noteworthy that the above models were framed in the context of associative learning, in which the predictive structure of the stimuli is directly relevant to the task, being typically associated to rewards or punishments. However, learning can also occur in situations in which it is not useful to the task. In the context of perceptual learning (i.e., the enhancement of perceptual performance consecutive to repeated stimulation; Lu et al., 2011), the importance of attention remains controversial. On the one hand, Watanabe et al. (2001) found that subthreshold task-irrelevant stimuli affected the discrimination of supra-threshold stimuli, thus suggesting that attention is not needed for perceptual learning to occur. On the other hand, other studies showed opposite results: only task-relevant and actively attended information was learnt (Shiu and Pashler, 1992; Ahissar and Hochstein, 1993). In the context of “statistical learning”, a type of learning first coined by Saffran et al. (1996) in the framework of language acquisition in infants, statistical environmental contingencies can also be learned in situations in which they are not useful to the task (Zhao et al., 2013), and may sometimes remain entirely implicit (Saffran et al., 1999; Fiser and Aslin, 2001; Perruchet and Pacton, 2006). Statistical learning occurs also irrespective of the perceptual modality; indeed, it has been reported in the visual (Turk-browne et al., 2005, 2008), auditory (Saffran et al., 1999) and tactile domains (Conway and Christiansen, 2005). Despite a wealth of studies investigating the general mechanisms of statistical learning, its relation to attention remains poorly understood. Previous studies have suggested that attentional allocation is necessary for the learning to occur (Toro et al., 2005; Turk-browne et al., 2005). Conversely, only one study has addressed the effect of statistical learning on attentional allocation (Zhao et al., 2013), arguing in favor of the hypothesis that statistical regularities attract attention, even when not task-relevant. However, in that study, only covert attention allocation was assessed, during a task in which statistical regularities were always irrelevant to the task being performed.

In the current study, we addressed the issue of the relationship between visual attention and statistical learning when the statistical structure of the stimulus sequence is either relevant to the task or not. We performed an experiment in which statistical regularities were manipulated during a simple color detection task, while recording eye movements. Specifically, we controlled the conditional probability of occurrence of the different colors, such that some colors allowed predicting the target occurrence whereas the others did not. We evaluated statistical learning by measuring reaction times (RTs) as a function of color predictability (Abla and Okanoya, 2009; Barakat et al., 2013), while visual attention allocation was estimated by comparing the position, velocity and acceleration of the eyes with respect to those of the stimuli. The aim of the current study was to provide evidence in favor of one or the other aforementioned model, by comparing the attentional allocation to both the predictive and predicted stimuli in trials where a target was present or not.

## Materials and Methods

### Participants

Nineteen healthy participants (mean age = 24.4, SD = 2.98, 12 females) took part in the experiment. All of them reported normal or corrected-to-normal vision. The experiments were carried out according to the Declaration of Helsinki and were approved by the Ethics Committee of the Université catholique de Louvain. Written informed consents were obtained from all the participants.

### Experimental Design and Equipment

The experiment took place in a dim and quiet room, and lasted for around 40 min. The participants were seated comfortably on a chair in front of a 19″ CRT screen, with a 75 Hz refresh rate, with their head resting on a chinrest 58 cm from the screen to ensure stability during the eye-tracking recordings. An Eyelink^©^ 1000 + eye tracker (SR Research Ltd., Kanata, Ontario, Canada) monitored eye movements and blinks at a sampling frequency of 500 Hz. The task was implemented using the version 3.0.9 of the Psychotoolbox (Brainard, 1997) with Matlab 7.5 (The MathWorks, Natick, MA, USA).

The experiment consisted of a color detection task: the participant had to click on a computer mouse with the right index finger whenever one of the two dots (1° wide) moving on the screen featured the target color. The experiment was composed of eight blocks lasting 4 min each. Between the 4th and 5th block, the participants were allowed to have a few minutes’ break, during which the light was turned on. Blocks were composed of 108 trials, and a different target color (*n* = 6) was designated every 18 trials, by displaying a large dot (4° wide) of that color at the center of the screen for 1500 ms. During each block, each of the six possible dot colors was used as target color, in randomized order. Each correct detection was signaled by a positive auditory feedback and associated to a monetary reward of 2 cents, while each wrong answer was associated with a negative auditory feedback and a negative reward of −2 cents.

**Figure 1:**
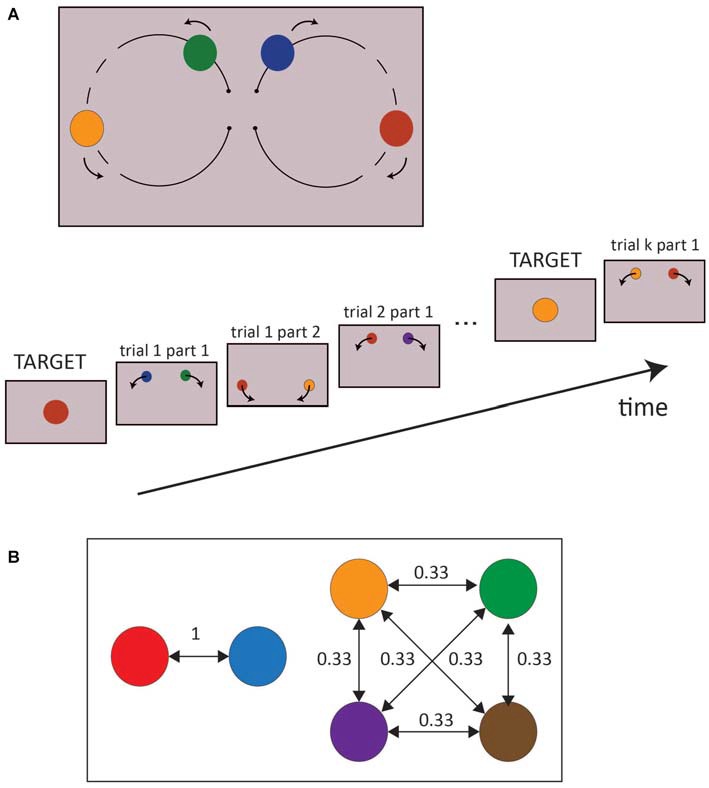
**(A)** Experimental Design. The upper part is a schematic representation of a whole trial, while the lower part of the picture represents the successive stages of a block. The dashed line represents the range of dot positions in which a change of color may occur. **(B)** An example of transition probabilities between colors is represented. Two colors were always associated to each other (conditional probability = 1, predictable colors) while the remaining colors all shared a conditional probability equal to 0.33 (unpredictable colors). The transition probabilities of the colors were pseudo randomized between subjects.

In each trial, the two colored moving dots were displayed over a gray background (70% of the maximum luminance of the screen) and moved along two circular paths (16° wide), starting from the center of the screen and heading upwards, such that the right dot moved clockwise toward the right part of the screen, and the left one anticlockwise to the opposite side (Figure 1A). The two dots always had the same velocity (180°/s), which was kept constant across trials; the total duration of the circular displacement of the colored dots was then 2000 ms. When the two moving dots were about midway, between 700–1500 ms after trial onset (randomized across trials), they both changed color simultaneously. Trials were separated by a 300 ms interval during which the screen remained gray. This particular design, i.e., the circular motion of the stimuli, was chosen to elicit spontaneous eye movements while driving the subject to fixate the center of the screen between each trial, even though no fixation cross was displayed. The six possible colors of the dots were red, blue, orange, brown, green and purple, and the two dots never had the same color.

Importantly, the probability distribution of the colors appearance on the screen was not random. In the first part of the trial, i.e., before the dot changed color, all the colors (including the targets) appeared with the same probability, but in the second part of the trial, the colors were conditional on the colors displayed in the first part. In particular, the colors were randomly split into two groups: two colors, selected pseudo-randomly between participants, predicted each other with a probability of 100%, while the remaining four colors predicted each other with a probability of 33% (Figure 1B). Moreover, the colors had the same overall frequency of occurrence, both in the first and second part of each trial. At the end of the experiment, participants were verbally asked if they had noticed any difference between the first and second parts of the trial, the left and right spots, and between colors. None of them reported any explicit bias in the experiment.

### Data Analysis

#### Reaction Time Analysis

First, we log-transformed the RTs in order to make their distribution closer to normal and removed the outliers (over ±3 SD, around 1% of the data). Second, since we found that the timing (700–1500 ms) of the color change affected the RTs for color detection in the second part of the trial (negative correlation between RTs and color change delay: *R* = −0.35, *p* < 0.0001), we removed this effect by considering the residuals of this regression. So the generalized linear mixed models (GLMM) analyses described below included as dependent variable the difference between the RT data and the predicted values obtained from the regression, effectively removing this influence from the data. There was no such effect in the first half of the trials.

#### Eye Movement Analysis

**Figure 2:**
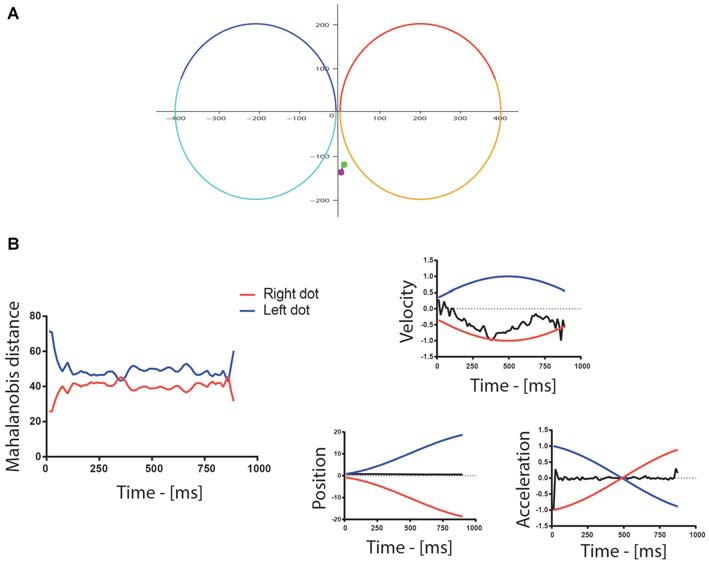
**(A)** Example of eye movements during a trial: the colored spots represent the beginning (green) and end (purple) of the eye trace. In this particular example, the position of the eyes changed little throughout the trial. The red/orange and the blue/cyan traces are the paths of the two dots for the first/second part of the trial. **(B)** The graph to the left shows the Mahalanobis distance between the eyes and the dots following the left (blue) and right (red) trajectories/paths in the first half of the trial. The Mahalanobis distance with the two dots was relatively large in this particular trial (around 42) because the position and acceleration profiles of the eyes differed strongly from the ones of the dots, as shown on the graphs on the right of the figure, which illustrate the normalized velocity, the position and the normalized acceleration of the dots (blue—left, red—right) and gaze (black). The position is reported in pixel, whereas the trial-wise normalization of velocity and acceleration was performed by dividing all values by the maximum of their absolute value. The velocity of the gaze, however, followed more closely the velocity of the right dot, which indicated attention allocation to the right in this example trial.

By inspecting the data we determined that taking into account only the distance between the eye and stimulus positions was not an effective way to determine attention allocation. As shown in Figure 2A, in trials in which the participants did not make a saccade toward one of the stimuli, the mere distance between the eyes and the two dots was poorly informative in terms of the actual attentional allocation. In contrast, comparing also the velocity and acceleration of the eyes with those of the targets (Figure 2B) revealed more accurately on which dot attention was allocated. This is consistent with the observation that subjects can track moving stimuli while maintaining their gaze confined in a narrow area and away from the stimuli (Hafed et al., 2008). Therefore, we combined all these measures in a single value, namely the Mahalanobis distance (Mahalanobis, 1936; De Maesschalck et al., 2000), to determine which dot was being tracked by attention. Specifically, we computed the Mahalanobis distance between the position, velocity and acceleration of the eyes and those of each of the two dots. A binary variable representing attentional allocation to the right/left was computed, assigning a value of one when the distance to the right/left dot was smaller than the distance to the left/right dot. This variable was then aligned either on the trial onset, or on the color change occurring around the middle of the trial and downsampled to 10 Hz in order to limit the number of time bins to analyze. For each 100 ms time bin, the total number of trials in which attention was allocated to the left/right dot was computed and used as a binomial variable in GLMM.

#### Statistical Methods

All the analyses were performed with the SAS 9.3 Software (SAS Institute, Cary, NC, USA), by means of GLMM. We ran GLMM on log-transformed RT data, modeled as a normally distributed variable. In this case the variables included were PREDICTABILITY, differentiating the predicted from the non-predicted colors, PART, which considered if the target was in the first or in the second part of the trial, and BLOCK, a continuous variable from 1–8. The SUBJECT factor was considered in the random models, along with all the other factors.

We ran several GLMM on the eye position data as well. In all of them, eye position was modeled as a binomial variable (see above). In the first GLMM we analyzed whether attention was more likely to be allocated on the target dot in trials where it was present (see Figure 4A). This analysis was performed mostly to confirm the validity of the Mahalanobis distance used in the present study, since we expected a strong attentional bias toward the target dot. The explanatory variables were TARGET-SIDE and TIME-BINS, the first one being representative of the position of the target (right or left side), the second one indicating the bin order (from 1 to 5, i.e., from 0 to 500 ms following stimulus display for the first half, and following color change for the second half). The residual covariance structure of the successive time bins was modeled with different variance parameters for each bin (variance component), in order to account for the correlations between successive bins. Because of limitations due to lack of convergence of the fitting algorithm used to optimize the GLMM, these analyses were performed separately for the first and second PART of the trials. In the second GLMM, we evaluated the allocation of attention to the predictive color when displayed together with a non-predictive color, while none of the stimuli were targets (see Figure 4B). The dependent variable in this case was the attentional allocation to the predictive color, and the independent variables were SIDE and TIME-BINS (same convention as before). Finally, in the last GLMM, we compared the trials in which a target shown in the second part of the trial was either predicted or non-predicted, while non-predictive colors were displayed on the other side. In this case the dependent variable indicated attentional allocation to the target, and the explanatory variables were PREDICTABILITY and TIME-BINS (from 1000 ms before the color change to 500 ms after the color change; see Figure 4C).

Finally, in order to investigate the timing of the effect of the color predictability, we also performed analyses on RT and attentional allocation restricted to the first block of the experiment, in which we split the first block in three sub-blocks of 36 trials each. Here the dependent variable was either the log-transformed and subject-wise normalized RT or the average of the attentional allocation variable over the whole duration of the first half of the trial. The explanatory variables were PREDICTABILITY and SUB-BLOCK.

## Results

### Reaction Time Analysis

**Figure 3:**
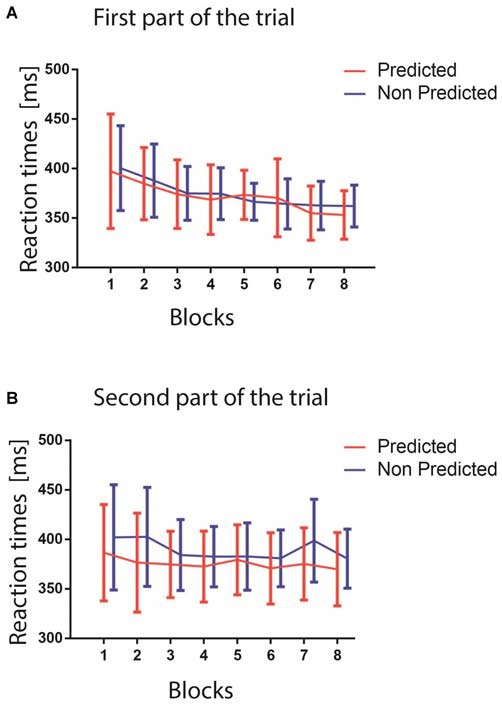
**(A)** The upper figure shows the reaction times (RTs) in ms for the two predictability conditions in the first part of the trials (red predicted colors, blue unpredicted colors). The blocks are represented on the *x*-axis. **(B)** The lower figure shows RTs as a function of the blocks for the second part of the trials (red predictable colors, blue unpredictable colors). In both panels, error bars represent standard errors of the mean computed within each block.

The results of the GLMM analysis on RTs revealed a significant main effect of the factors PREDICTABILITY (*F*_(1,18)_ = 5.46, *p* = 0.0313), and BLOCK (*F*_(7,126)_ = 6.90, *p* < 0.0001). Specifically, the BLOCK effect consisted of a decrease in the RTs across blocks, as showed in Figure 3 (Figure 3A shows RT from the first part of the trial, Figure 3B from the second part), while the PREDICTABILITY effect consisted of faster responses for predicted targets. Although the PART factor revealed no significant difference between the first and second part of the trial (*F*_(1,18)_ = 1.30, *p* = 0.2683), the interaction between the factors PREDICTABILITY and PART was significant (*F*_(1,18)_ = 6.20, *p* = 0.0228), revealing, as confirmed by pairwise comparisons, a difference between the predicted and non-predicted colors only in the second part of the trials (Figure 3B; *t*_(1,18)_ = 3.14, Tukey-Kramer adjusted *p* = 0.0266). This last result confirmed that the participants learnt implicitly the color association between the stimuli displayed in the first and second parts of the trials, and that learning this association helped them to react faster to the occurrence of the target color when appearing in the second part. The lack of significant interaction between BLOCK and PREDICTABILITY (*p* > 0.1) suggested that statistical learning occurred very early during the experiment, as already reported in a previous study (Turk-browne et al., 2009).

### Attention Allocation Analysis

**Figure 4:**
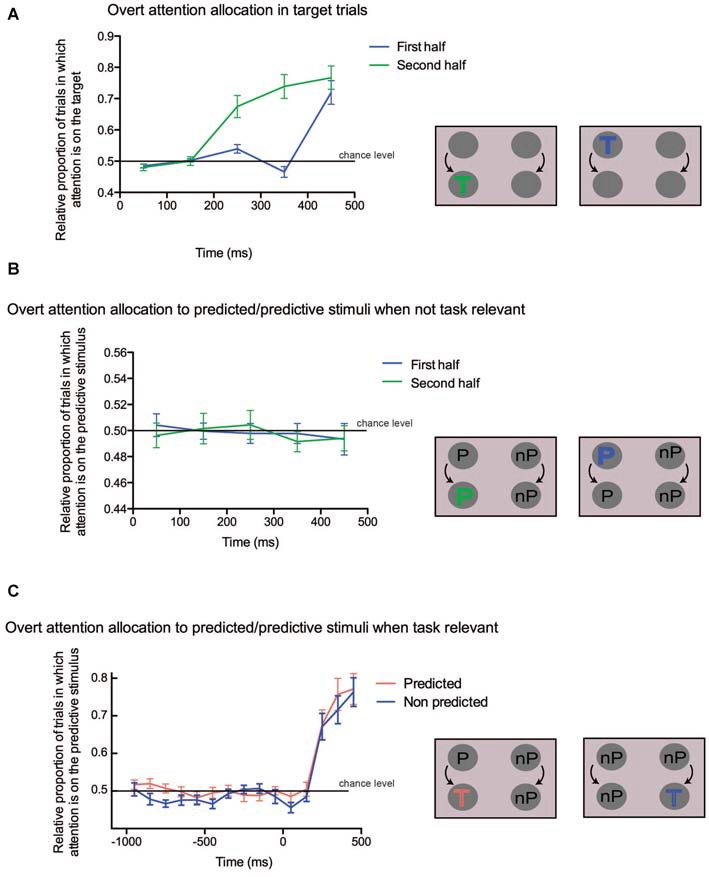
**(A)** Overt attention allocation for the trials in which a target occurred either in the first half (blue line) or in the second half (green line) of the trial. Time is shown along the *x*-axis, the proportion of trials in which targets attracted attention is on the *y*-axis. The 0 value on the *x* axis corresponds either to the beginning of the trial (first half) or to the change of color (second half). A schematic depiction of the analyzed conditions is shown on the right. The target could appear either in the first part (blue “T”) or in the second part (green “T”). **(B)** Overt attention allocation to predicted/predictive stimuli when not task-relevant. On the *x*-axis time is in ms, on the *y*-axis the proportion of trials in which predictive colors attracted attention is shown, indicated as the difference between the predicted and the non-predicted dots. A value of 0 on *x*-axis corresponds either to the beginning of the trial (first half) or to the change of color (second half). On the right, the predicted (“P”) and non predicted (“nP”) conditions are illustrated. **(C)** Overt attention allocation to the predicted stimulus when task relevant. On the *x*-axis time is in ms, while on the *y*-axis there is the relative proportion of trials in which attention is captured by the predictive stimulus when the target is either predictive or not. A value of 0 on the *x*-axis corresponds to the time of the change of color. The two conditions are illustrated on the right of the panel: in the first case the target is preceded by a predictive color (red “T”), whereas in the second case the target is preceded by a non-predictive color (blue “T”).

As expected, when analyzing target trials, we found a progressive allocation of attention to the target (see Figure 4A; main effect of TIME-BINS: first PART: *F*_(4,72)_ = 27.31, *p* < 0.0001; second PART: *F*_(4,72)_ = 41.38, *p* < 0.0001), irrespective of whether it was displayed on the left or right side of the screen (main effect of TARGET-SIDE: first PART: *F*_(1,18)_ = 2.53, *p* = 0.13; second PART: *F*_(1,18)_ = 0.87, *p* = 0.36; interaction: first PART: *F*_(4,72)_ = 0.55, *p* = 0.70; second PART: *F*_(4,72)_ = 0.62, *p* = 0.65). Tukey-corrected pairwise comparisons for the first part of the trial showed that the preferential allocation of attention to the target was significant only for the last bin (all comparisons between 5th bin and other bins: *p* < 0.001), whereas for the second part, all pairwise comparisons were significant (*p* < 0.05) except between the 1st and 2nd, 3rd and 4th and 4th and 5th bins. Overall, these results highlight the attentional capture by the target both in the first and second part of the trial, confirming the validity of the attention allocation measure used in the current study. Moreover, they suggest that participants were slower in allocating attention to the target in the first part of the trial (5th bin, around 450 ms) than in the second part (from the 3rd bin on, starting around 250 ms). However, we cannot exclude that this unexpected discrepancy could also be explained by the fact that, early in the first part of the trial, targets were closer from each other than during the second part of the trial. This larger proximity between the stimuli could have hampered the sensitivity of our measure to detect differential attentional allocation to the target side.

We then looked at the allocation of attention to the predictive stimuli when no target was present on the screen. We failed to find any significant change in attentional allocation to the predictive stimulus over time (see Figure 4B; main effect of TIME-BINS: first PART: *F*_(4,72)_ = 0.07, *p* = 0.99; second PART: *F*_(1,18)_ = 0.13, *p* = 0.7196), irrespective of the stimulus side (main effects and interactions with the SIDE factor: all *p* > 0.15). The intercept of the model, representing the overall allocation of attention on the predicted stimulus irrespective of both TIME-BINS and SIDE was not significantly different from zero (*p* > 0.7 in both PARTS). These results suggest that regularities, when they were not instrumental to the task at hand, did not capture attention.

Finally, we compared trials in which a target appearing in the second part was either predicted or not by the color shown in the first part of the trial. Here the TIME-BINS factor included 15 bins, ranging from 1000 ms before to 500 ms after target onset. Since the target could appear as early as 750 ms after the trial onset, the bins ranging between −1000 ms and −700 ms contained slightly fewer trials than the later bins. We found that attention was preferentially allocated to the target when it was predicted (main effect of PREDICTABILITY: *F*_(1,18)_ = 4.81, *p* = 0.042; see Figure 4C). There was also a significant effect of TIME-BINS (*F*_(14,252)_ = 14.56, *p* < 0.0001), merely indicating the progressive allocation of attention to the target location, but no significant interaction (*F*_(14,252)_ = 0.21, *p* = 0.99). This lack of interaction suggested that attention might have been driven towards the predictive stimulus already in the first part of the trial. In order to confirm this hypothesis, we tested the same model but restricted to the first part of the trials (i.e., the first 8 bins) and still found a significant PREDICTABILITY effect (*F*_(1,18)_ = 5.71, *p* = 0.028), with no significant interaction (*F*_(7,126)_ = 1.43, *p* = 0.20). This confirms that attention was biased during the first part of the trial toward the colored dot, which predicted target occurrence in the second part.

### Temporal Dynamics of Learning and Selective Attention in the First Block

**Figure 5:**
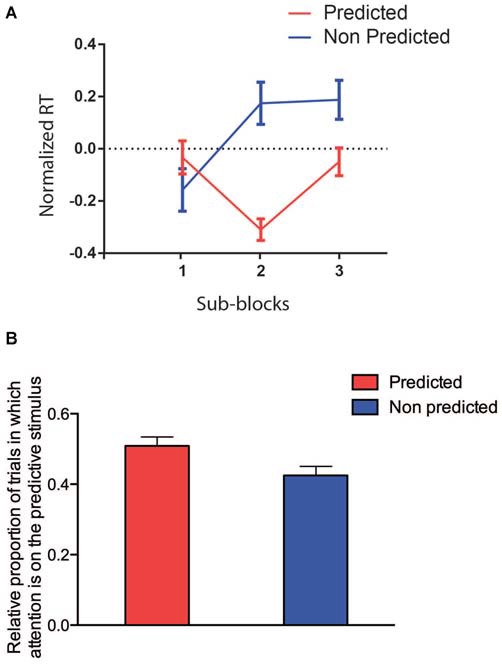
**(A)** RTs restricted to the first block (red predictable colors, blue unpredictable colors). On the *x*-axis, the three sub-blocks are, on the *y*-axis the log-transformed and normalized RTs are represented. **(B)** Attention allocation restricted to the first block (red predictable colors, blue unpredictable colors).

In order to determine when statistical learning effects started to influence the RT, we performed a GLMM restricted to the data from the first block only and considering the SUB-BLOCK (*n* = 3, 36 trails each) and PREDICTABILITY as factors. We found no main effect of SUB-BLOCK (*F*_(2,29)_ = 0.67, *p* = 0.5193) or PREDICTABILITY (*F*_(1,17)_ = 3.80, *p* = 0.0678), but a significant interaction between these two factors (*F*_(2,251)_ = 5.31, *p* = 0.0055). The difference between the two PREDICTABILITY conditions became significant from the second sub-block (see Figure 5A, Tukey-corrected pairwise comparisons, all *p* ≤ 0.05; *t*_(251)_ = 3.10, *p* = 0.0260). In Conclusion, around 72 trials seem to be enough to learn the color association between predictive and predicted colors.

Finally, we analyzed the effect of PREDICTABILITY on the allocation of attention to the target, restricted to the first block (and to time bins preceding the target onset; Figure 5B). We found again only a significant effect of PREDICTABILITY (*F*_(1,18)_ = 7.15, *p* = 0.015), showing that the preferential allocation of attention to the predicted location of the target occurred very early.

## Discussion

In the current study we investigated the influence of statistical regularities on the allocation of visual attention in a color detection task. To do so, we manipulated the conditional occurrence of a sequence of colored stimuli, while recording the RT and eye position. None of the participants reported any awareness of the conditional occurrence manipulation, suggesting that it remained implicit during the whole experiment. As previously reported in the literature (Turk-browne et al., 2005; Dale et al., 2012), despite being implicit, the temporal predictability of the targets affected markedly the behavioral results, as revealed by shorter RTs in the detection task. Strikingly though, we found that this behavioral advantage was already measurable after a few dozen of trials, proving its remarkable efficacy.

Regarding visual attentional allocation, we report that predictability biases attention only when regularities are instrumental to the execution of the task. In other words, attention was biased by the regularities only when the target was predicted, whereas when the predicted stimulus was not the target, and therefore the statistical structure of the sequence was not helpful to perform the task, both predictive (first part of the trial) and predicted stimuli (second part of the trial) failed to either attract or divert attention. These findings are in agreement with the model proposed by Mackintosh (1975), in which predictable stimuli, when either rewarding or related to the behavior of the agent, attract attention.

In the context of the associative learning field, attention is considered as having different possible functions (Gottlieb, 2012): attention for learning, attention for action and attention for liking. Attention for learning drives attention towards uncertain stimuli, in order to learn new regularities in the environment. Attention for action refers to the allocation of attention in order to optimize the achievement of a certain goal, while attention for liking is attracted to pleasurable and rewarding stimuli. Our finding that regularities attract attention, when they predict the target appearance, is in accordance with the concept of attention for action. On the other hand, predictive stimuli did not attract attention when they were predicting the occurrence of a distractor, even though they allowed inferring that the target would not appear on that side. This may be explained by the implicitness of the learning, which could have prevented participants to make such inference (Custers and Aarts, 2011). The attentional allocation to stimuli predicting the target appearance is also in accordance with attention for liking, since finding the target was always associated to a reward.

Regarding attention for learning, we found that, when focusing on the first block, only 72 trials were necessary for the RT effect to emerge. Similarly, the bias in visual attention toward the predictive stimuli reached significance in the first block as well. These findings seem to indicate that both effects appeared concurrently, in apparent contradiction with the hypothesis that attention would be biased towards potential sources of relevant information such as statistical regularities before any behavioral advantage emerges (Hogarth et al., 2008; Holland and Maddux, 2010), thus being causal in the development of this advantage. This suggests instead that both the faster detection of predicted targets and the preferential allocation of attention to the predicted targets are part of the same underlying statistical learning phenomenon. Obviously, it could also be argued that no temporal dissociation between the attentional allocation and the learning processes was found in the present study because of a lack of sensitivity either of our design or of our measurement method. Possibly, a more complex statistical design, requiring a longer learning time, could allow us to dissociate the time course of the attentional allocation from the behavioral signature of learning.

Nevertheless, how this statistical learning process develops during the early stage of the task, either before or after attention becomes preferentially allocated to the statistical structure, remains unanswered. It could be proposed that statistical learning occurs pre-attentively, i.e., in the absence of attentional allocation (Li, 2000; Zénon et al., 2009a). But since attention is thought to be necessary for the learning of the statistical regularities to occur (Turk-browne et al., 2005), it is likely that during the early phase of the task, learning takes place when, thanks to the random exploration of the scene by visual attention, attention is allocated by chance to the relevant location.

In the context outlined by our design, it seems that attention allocation is mainly biased by the optimization of the task’s performance. This is in accordance with past computational studies proposing that visual selection is a mechanism involved in solving the inference problem of predicting the evolution of the environment to optimize task performance (Dayan et al., 2000; Kruschke, 2003). However, this conclusion is at odds with the one from a recent study showing that, even when irrelevant to the task, regularities attracted covert attention (Zhao et al., 2013). It is noteworthy that in Zhao’s study, the difference between attentional allocation on informative and non-informative stimuli was small, and most importantly, the regularities were present in a stimulus feature that was irrelevant to the task. In contrast, in our experiment, regularities involved the main feature used during the task, even when no target was present, i.e., the color of the dots. In addition, Zhao et al. (2013) investigated specifically covert attention by looking at discrimination performance while participants maintained their gaze on a central fixation point. In contrast, we evaluated attentional allocation by looking at eye position, speed and velocity with respect to stimulus position, speed and velocity. This novel measure of attention allocation does not allow to dissociate overt from covert attention because, even tough, it relies on eye movements, it measures also attentional allocation to peripheral stimuli. In some instances, our measure indexes the fixation of gaze on one of the targets (i.e., overt attention), whereas in other cases we highlight attentional allocation to one specific peripheral location while the gaze remains fixed on a central position, corresponding to covert attention (Filali-Sadouk et al., 2010; Zénon et al., 2014). The choice of using the eye velocity and acceleration signals, in addition to their position, to track the allocation of attention, was driven by the observation that observers can track moving stimuli while maintaining their gaze away from them. As reported in a study by Hafed et al. (2008), non-human primates are able to track an imaginary point located between two moving stimuli while maintaining the moving stimuli in peripheral vision. In our experiment, as illustrated in Figure 2, even when the position of the eyes was kept at the center of the screen, the velocity profile revealed clearly that the subject was in fact actively tracking one of the two dots, thus revealing preferential attentional allocation. We confirmed the validity of our approach based on velocity and acceleration in the present study by showing, as expected, a strong bias to the target stimulus when displayed on the screen. More experiments will be needed to determine whether the discrepancy between our findings and the ones reported in the study of Zhao et al. (2013) is caused by the different attentional measures (covert vs. overt) or by the task-relevance of the visual features guiding attention.

To summarize, this study shows how implicit learning, as many other cognitive processes (Graf Estes et al., 2007; Brady et al., 2009; Zénon et al., 2009b; Umemoto et al., 2010), affects visual attention in a target detection task. When stimuli become predictable through the manipulation of conditional occurrences, these statistical regularities are learned very rapidly, and visual attention gets attracted to the informative stimuli when they are instrumental to the task at hand.

## Author Contributions

All authors listed, have made substantial, direct and intellectual contribution to the work, and approved it for publication.

## Conflict of Interest Statement

The authors declare that the research was conducted in the absence of any commercial or financial relationships that could be construed as a potential conflict of interest.
